# Dental and skeletal effects of combined headgear used alone or in association with rapid maxillary expansion

**DOI:** 10.1590/2177-6709.20.5.043-049.oar

**Published:** 2015

**Authors:** Milton Meri Benitez Farret, Eduardo Martinelli de Lima, Marcel M. Farret, Laura Lutz de Araújo

**Affiliations:** 1 Professor of Orthodontics, Undergraduate Program, Universidade Federal de Santa Maria, Santa Maria, Rio Grande do Sul, Brazil; 2 Adjunct professor of Orthodontics, Pontifícia Universidade Católica do Rio Grande do Sul (PUC/RS), Porto Alegre, Rio Grande do Sul, Brazil.; 3 PhD in Orthodontics, Pontifícia Universidade Católica do Rio Grande do Sul (PUCRS), Porto Alegre, Rio Grande do Sul, Brazil.; 4 MSc in Orthodontics, Pontifícia Universidade Católica do Rio Grande do Sul (PUC/RS), Porto Alegre, Rio Grande do Sul, Brazil

**Keywords:** Cephalometry, Extraoral traction appliance, Angle Class II malocclusion

## Abstract

**Objective:**

The aim of this study was to assess the effects of combined headgear used alone or in association with rapid maxillary expansion, as the first step for Class II malocclusion treatment.

**Methods:**

The sample comprised 61 patients divided into three groups: Group 1, combined headgear (CH); Group 2, CH + rapid maxillary expansion (CH + RME); and Group 3, control (CG). In Group 1, patients were treated with combined headgear until Class I molar relationship was achieved. In Group 2, the protocol for headgear was the same; however, patients were previously subject to rapid maxillary expansion.

**Results:**

Results showed distal displacement of maxillary molars for both experimental groups (*p* < 0.001), with distal tipping only in Group 1 (CH) (*p* < 0.001). There was restriction of forward maxillary growth in Group 2 (CH + RME) (*p* < 0.05) and clockwise rotation of the maxilla in Group 1 (CH) (*p* < 0.05).

**Conclusion:**

Based on the results, it is possible to suggest that treatment with both protocols was efficient; however, results were more significant for Group 2 (CH + RME) with less side effects.

## Abstract (pt)

**Objetivos:**

a finalidade do presente estudo foi verificar os efeitos do aparelho extrabucal de tração combinada, associado ou não à expansão rápida da maxila, como primeiro passo do tratamento da má oclusão de Classe II.

**Métodos:**

a amostra consistiu de 61 indivíduos divididos em três grupos: Grupo 1 = extrabucal de tração combinada (TC); Grupo 2 = tração combinada + expansão rápida da maxila (TC +ERM); Grupo 3 = controle (GC). No Grupo 1 os indivíduos foram tratados com extrabucal de tração combinada até a relação molar ficar em Classe I. No Grupo 2, o protocolo foi o mesmo, entretanto, os indivíduos receberam, previamente, expansão maxilar rápida.

**Resultados:**

os resultados mostraram deslocamento distal dos molares superiores em ambos os grupos experimentais (*p* < 0,001), com inclinação distal somente no Grupo 1 (TC) (*p* < 0,001). Houve restrição de crescimento anterior da maxila no Grupo 2 (TC + ERM) (*p* < 0,5) e rotação horária da maxila no Grupo 1 (TC) (*p *< 0,5).

**Conclusão:**

baseado nos resultados, é possível concluir que o tratamento com ambos os protocolos foi eficiente; entretanto, os resultados foram mais significativos para o Grupo 2 (TC + ERM), e com menos efeitos colaterais.

## INTRODUCTION

Class II malocclusion can result from multiple combinations of dental and/or skeletal relationships established between the maxilla and mandible.^1^ Headgear followed by the use of full fixed orthodontic appliance can be considered the gold standard treatment for children and adolescents with skeletal Class II malocclusion.^2^ Extraoral forces hold maxillary forward displacement while the mandible grows forward naturally. Since the 1950s, orthodontists have used headgears successfully and produced favorable dental and orthopedic effects proved by cephalometric analysis.^3^ There is scientific evidence that headgear can reduce facial convexity and improve the sagittal relationship between upper and lower dental arches.^4^^-^ 7

The morphological characteristics of Class II malocclusions usually include transverse maxillary deficiency.^8^^,^ 9 ^,^ 10 In those cases, patients should undergo rapid maxillary expansion.^9^^,^ 10 ^,^ 11 According to Haas^10^ and Lima Filho et al,^9^ there is marked upper arch constriction in the region between canines in individuals with Class II, Division 1 malocclusion. Maxillary constriction should be corrected by rapid maxillary expansion, followed by the use of headgear whenever necessary. Headgear appliances provide different force systems according to the direction of traction.^12^ Cervical headgear is generally indicated for patients with hypodivergent facial types, while high-pull headgear is more commonly used in hyperdivergent faces.^13^^-^ 16 Nevertheless, combined headgear has been used in a wide variety of cranial-facial architetures.^17^

From the clinical orthodontist's standpoint, the question is whether the benefits of rapid maxillary expansion before combined traction headgear is used are really worth it when treating Class II malocclusion. Therefore, the aim of this study was to assess maxillary dental and skeletal effects caused by combined headgear used alone or in association with rapid maxillary expansion in adolescents with Class II, Division 1 malocclusion. 

## MATERIAL AND METHODS

The experimental sample comprised 41 individuals (18 boys and 23 girls) with Class II, Division 1 malocclusion, aged between 9 and 13 years old and treated by combined headgear (CH) as the first step of orthodontic treatment. A total of 20 individuals (8 boys and 12 girls) with Class I malocclusion were assessed during the development of dentition and served as controls.

Research subjects were selected from the records of 400 individuals available in the files of the Clinic of Orthodontics, School of Dentistry, Pontifícia Universidade Católica do Rio Grande do Sul, Brazil. All treated and control individuals had good general and oral health conditions, were in the pubertal growth period, and had less than 3 mm of crowding in the lower arch. The research was approved by the university Institutional Review Board (10/05127). 

Initial records (T_1_) included patient's medical and dental history, dental casts, and Lateral cephalograms. Dental casts determined the diagnosis of Class II malocclusion associated or not with transverse maxillary deficiency. In Class II, first molars should at least present a cusp-to-cusp relationship. Transverse maxillary deficiency was determined when the distance between maxillary molars was 4 mm less than the distance between mandibular molars, as described previously.^16^ Based on anteroposterior and transversal first molar relationship, subjects were allocated into Group 1 (Class II, normal transverse maxilla) or Group 2 (Class II, transverse maxillary deficiency). Group 3 comprised control individuals with Class I molar relationship and normal transverse maxilla.

Subjects in Group 1 (n = 20, 8 boys and 12 girls) had Class II malocclusion with normal transverse maxilla and were treated with combined headgear (CH), 12 to 14 hours per day, during six months. The headgear outer bow was parallel to the inner bow and had hooks in the region of first molars. The inner bow was expanded 2 mm before being inserted into the molar tubes. Forces of 300 g/f were applied in both parietal and cervical direction on each side. The equation V_r _= √ V_c_ 2 + V_p_ 2, in which V_r _is the resultant vector, V_c _the cervical vector, and V_p _is the parietal vector, established that the resultant vector was equal to 424 f/g. Subjects allocated in Group 2 (n = 21, 10 boys and 11 girls) had Class II malocclusion associated with transverse maxillary deficiency. Thus, before headgear therapy, patients underwent rapid maxillary expansion (RME + CH) during 14 days. A modified Haas expander, banded to first molars and bonded up to first premolars or first deciduous molars, was activated four times a day on the first day and twice a day thereafter, until transverse overcorrection was achieved. On the seventh day of expansion, patients started the 6-month therapy with combined headgear, following the same protocol applied for Group 1 (CH). In Group 3, control subjects (n = 20, 8 boys and 12 girls) had Class I malocclusion with normal transverse maxilla. During the 6-month period of the study, they underwent space supervision procedures only, including space maintenance or wearing of deciduous teeth.

At baseline, cephalometric measurements showed that all groups were representative of slightly hyperdivergent individuals. Mandibular plane angle (SN.GoGn) was 36.9 ± 3.9^o^ in Group 1 (CH), 36.4 ± 6.3^o^ in Group 2 (RME+CH) and 36.9 ± 4.1^o^ in Group 3 (control). On the other hand, ANB angle highlighted a Class II skeletal pattern in the treated groups (CH = 5 ± 1.9^o^, RME+CH = 5.9 ± 1.8^o)^ and a Class I skeletal pattern in the control group (3.7 ± 2.2^o)^. 

**Figure 1:**
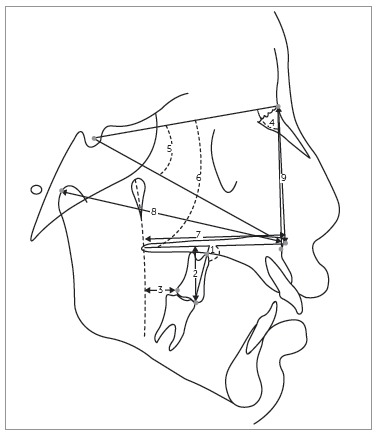
- Anatomical tracing and cephalometric measurements. Dental measurements: molar inclination (1), molar height (2) and anteroposterior molar (3). Maxillary measurements: SNA (4), SN.Ptm-Sn (5), SN.PP(6), Ptm-Sn (7), Co-Sn (8) and N-Sn (9).

Follow-up records (T_2_) of experimental groups (Group 1 [CH], Group 2 [RME+CH]) included lateral cephalograms taken when Class I molar relationship was achieved, on average, six months after headgear therapy onset. The follow-up records of Group 3 (control) were taken six months later, on average; similar to the experimental groups when Class I molar relationship was achieved. Cephalograms were manually taken in random order. Afterwards, the cephalometric landmarks were digitized with the aid of Dentofacial Planner Plus (DFP 2.0) software by an operator blind to subject and group. Cephalometric measurements were selected to assess dental and skeletal effects of treatment on the maxilla (Fig 1). Statistical analysis was performed by Student's t-test for comparison between T_1_ and T_2_ in each group. One-way analysis of variance (ANOVA) and Tukey's multiple comparison tests were applied to compare differences (T_2_−T_1_) between groups.

## RESULTS

### Molars

Distal movement of maxillary molars occurred in both experimental groups during the study period (*p* < 0.001), but distal tipping occurred only in Group 1 (CH) (*p* < 0.001) (Tables 1 and 2). However, the amount of distal tipping of maxillary molars did not differ whether the headgear was used alone or in association with maxillary expansion (*p* > 0.05) (Table 4). There was no extrusion of maxillary molars in either one of the experimental groups (*p* > 0.05).

**Table 1:** - Means, standard deviation, mean difference and Student's t-test comparing initial (T_1_) and control (T_2_) values in Group 1 (CH) (n = 20).

**Measurements**	**T_1_**	**T_2_**	**Mean difference**	***P*-value**		
	**Mean **	**SD**	**Mean **	**SD**		
Molars						
Molar inclination (degrees)	101.9	3.8	108.3	6.3	6.4	0.000***
Molar height (mm)	19.1	2.2	19.0	2.4	-0.1	0.66
Anteroposterior molar (mm)	-8.7	2.4	-6.4	2.3	2.3	0.000***
Maxilla						
SNA (degrees)	81.4	3.8	80.7	3.3	-0.3	0.24
SN.SSn (degrees)	23.4	1.6	24.0	1.6	0.5	0.02*
SN.PP (degrees)	10.5	2.1	11.7	2.3	1.2	0.001**
Ptm-Sn (mm)	51.1	3.4	50.7	2.9	-0.4	0.14
Co-Sn (mm)	88.4	5.1	89.7	5.2	1.2	0.01*
N-Sn (mm)	54.5	4.1	56.2	3.7	1.6	0.000***

* *p *< 0.05; ** *p *< 0.01; *** *p *< 0.001.

**Table 2:** - Means, standard deviation, mean difference and Student's t-test comparing initial (T_1_) and control (T_2_) values in Group 2 (CH + RME) (n = 21).

**Measurements**	**T_1_**	**T_2_**	**Mean difference**	***P*-value**		
	**Mean **	**SD**	**Mean **	**SD**		
Molars						
Molar inclination (degrees)	102.8	3.2	104.3	4.4	1.4	0.18
Molar height (mm)	21.0	2.3	21.5	2.2	0.4	0.07
Anteroposterior molar (mm)	-8.0	3.1	-6.5	2.9	1.4	0.001**
Maxilla						
SNA (degrees)	81.3	3.5	80.7		-0.6	0.02*
SN.SSn (degrees)	23.5	2.2	23.8	2.3	0.3	0.15
SN.PP (degrees)	9.7	2.8	10.3	2.8	0.5	0.06
Ptm-Sn (mm)	52.1	3.6	52.1	3.3	0.0	0.77
Co-Sn (mm)	89.7	4.6	89.8	4.7	0.1	0.75
N-Sn (mm)	54.0	2.5	55.2	2.8	1.1	0.001**

* *p *< 0.05; ** *p *< 0.01; *** *p *< 0.001.

**Table 3:** - Means, standard deviation, mean difference and Student's t-test comparing initial (T_1_) and control (T_2_) values in Group 3 (C) (n = 20).

**Measurements**	**T_1_**	**T_2_**	**Mean difference**	***P*-value**		
	**Mean **	**SD**	**Mean **	**SD**		
Molars						
Molar inclination (degrees)	102.2	3.6	102.8	5.9	0.6	0.62
Molar height (mm)	20.7	2.1	21.1	2.6	0.4	0.14
Anteroposterior molar (mm)	-8.4	2.8	-8.7	3.5	-0.2	0.60
Maxilla						
SNA (degrees)	80.0	3.5	79.9	2.9	-0.3	0.91
SN.SSn (degrees)	23.8	2.6	23.2	2.4	-0.5	0.18
SN.PP (degrees)	9.6	3.0	9.1	3.0	-0.4	0.25
Ptm-Sn (mm)	50.3	2.1	51.0	2.8	0.6	0.06
Co-Sn (mm)	86.4	4.0	87.2	4.1	0.8	0.01*
N-Sn (mm)	51.2	2.7	51.9	2.9	0.7	0.04*

* *p *< 0.05; ** *p *< 0.01; *** *p *< 0.001.

**Table 4:** - Minimum and maximum differences, means, standard deviation and one-way analysis of variance supplemented by Tuke's multiple comparisons test comparing groups at two intervals.

**Measurements**	**Groups**	**Difference (T_2 _- T_1_)**	**SD**	***P*-value**		
		**Minimum**	**Maximum**	**Mean**		
Molars						
Molar inclination (degrees)	Group 1 (CH)	-1.0	18.6	6.4^B^	5.2	0.002**
	Group 2 (CH + RME)	-11.9	8.0	1.4A^B^	5.0	
	Group 3 (Control)	-8.4	14.0	0.6^A^	5.7	
Molar height (mm)	Group 1 (CH)	-1.7	2.4	-0.1	1.1	0.22
	Group 2 (CH + RME)	-1.7	3.4	0.4	1.1	
	Group 3 (Control)	-1.6	2.8	0.4	1.1	
Anteriorposterior molar (mm)	Group 1 (CH)	-0.5	6.1	2.3^B^	1.6	0.000***
	Group 2 (CH + RME)	-2.2	3.7	1.4^B^	1.6	
	Group 3 (Control)	-3.3	6.6	-0.2^A^	2.4	
Maxilla						
SNA (degrees)	Group 1 (CH)	-2.3	1.5	-0.0	1.1	0.33
	Group 2 (CH + RME)	-2.6	1.4	-0.6	1.1	
	Group 3 (Control)	-1.7	4.2	-0.3	1.3	
SN.SSn (degrees)	Group 1 (CH)	-1.0	3.0	0.5^B^	0.9	0.02*
	Group 2 (CH + RME)	-2.0	3.0	0.3^AB^	1.1	
	Group 3 (Control)	-4.1	2.7	-0.5^A^	1.8	
SN.PP (degrees)	Group 1 (CH)	-1.0	4.0	1.2^B^	1.4	0.004**
	Group 2 (CH + RME)	-2.4	2.7	0.5^AB^	1.3	
	Group 3 (Control)	-3.4	2.8	-0.4^A^	1.6	
Ptm-Sn (mm)	Group 1 (CH)	-3.8	1.3	-0.4	1.3	0.5
	Group 2 (CH + RME)	-2.7	4.4	0.1	1.4	
	Group 3 (Control)	-1.9	4.0	0.6	1.5	
Co-Sn (mm)	Group 1 (CH)	-3.6	5.1	1.2	2.2	0.15
	Group 2 (CH + RME)	-3.3	5.0	0.1	1.9	
	Group 3 (Control)	-1.3	3.9	0.8	1.3	
N-Sn (mm)	Group 1 (CH)	-1.1	4.8	1.6	1.3	0.11
	Group 2 (CH + RME)	-1.2	4.4	1.1	1.3	
	Group 3 (Control)	-2.5	3.4	0.7	1.4	

* *p* < 0.05; ** *p*< 0.01; *** *p* < 0.001. Means followed by the same letter do not differ.

### Maxilla

Clockwise rotation of the maxilla occurred between T_1_ and T_2_ only in Group 1 (CH) (*p* < 0.05) (Table 1). Nevertheless, the values of maxillary clockwise rotation did not differ whether the headgear was used alone or in association with maxillary expansion (*p* > 0.05) (Table 4). There was restriction of forward maxillary growth between T_1_ and T_2_ only in Group 2 (CH + RME) (*p* < 0.05) (Table 2). However, the variation occurring in Group 2 did not differ from that found in Groups 1 and 3 (*p* > 0.05) (Table 4).

Class I molar relationship was achieved in 6.5 ± 1 months in Group 1 and 5.5 ± 1.1 months in Group 2. 

## DISCUSSION

The combined headgear is well indicated to treat patients with Class II malocclusion and mesodivergent or hyperdivergent facial patterns.^12^^,^ 14 ^,^ 15 On the other hand, cervical headgear is more suitable in cases of hypodivergent or mesodivergent facial patterns in which extrusion of maxillary molars would not hinder facial esthetics.^14^^,^ 18 ^,^ 20 Molar extrusion can cause clockwise rotation of the mandible and increase anterior facial height.^17^ High-pull headgear is usually recommended for cases of marked hyperdivergent facial pattern associated or not with anterior open bite.^15^^,^ 20 ^,^ 22

Transverse maxillary deficiency is often associated with Class II malocclusion, especially Class II, Division 1.^9^^,^ 11 ^,^ 16 Upper arch constriction in the region of canines may lead to mandibular retrognathism, which impairs natural anteroposterior growth of the mandible.^9^^,^ 11 Should transverse maxillary deficiency be diagnosed, rapid maxillary expansion should be carried out to maximize the benefits of orthodontic treatment for Class II patients.^8^^,^ 10

Mesodivergent and hyperdivergent facial patterns are predominant in cases of Class II, Division 1 malocclusion. However, the literature lacks evidence on the effects of combined headgear, associated or not with rapid maxillary expansion, over dentofacial structures. The present study analyzed the primary effects of combined headgear associated or not with rapid maxillary expansion, as the first step of comprehensive treatment of Class II malocclusions.

Follow-up records were taken when maxillary and mandibular first molars achieved Class I relationship. Despite the importance of assessment presented herein, further studies should include the final results of treatment. Cephalometric measurements were selected based on their potential to analyze the behavior of dental and skeletal maxillary structures. 

The design of the appliance followed standards adopted in a previous study,^16^ with the outer bow parallel to the inner bow and ending in the region of first permanent molars. The design of the headgear is strongly associated with its effects on maxillary molars. In combined headgears, longer and/or downward angled outer bows produce resultant forces that maximize vertical upward vectors, avoiding molar extrusion, but increasing distal tipping.^11^ On the other hand, cervical headgears with shorter outer bows would maximize the horizontal vectors, producing a resultant force in distal direction, which can reduce the tendency towards molar inclination, but still prevent extrusion.^16^ Although outer bows angled upward can eliminate molar inclination, this design may lead to undesirable extrusion of molars usually associated with clockwise rotation of the mandible, which jeopardizes Class II malocclusion treatment.^12^^,^ 16 ^,^ 22

Distal movement of maxillary molars was found occur in both Class II malocclusion treatment approaches. It was clear that combined headgear was effective in producing distal dental movement whether associated or not with maxillary expansion. Distal tipping of maxillary molars was found only in Group 1, which included individuals treated by headgear alone. In the present study, mean maxillary molars distal tipping was of 6.4 degrees in Group 1 (CH), very close to the value of 6.9 degrees found by Üçem and Yüksel.^22^ In Group 2 (CH+RME), molar distal tipping decreased to 1.4 degrees. Despite no statistical significant differences being found between groups, it seems that maxillary expansion was useful in preventing molar inclination. The connection of maxillary molars with the expander's acrylic plate and premolars would increase anchorage against distal tipping.^16^ There was no extrusion of maxillary molars either if the headgear was used alone or in association with the maxillary expander. Üçem and Yüksel have already showed that combined headgear avoided extrusion of maxillary molars.^22^This is a positive result, since molar extrusion would be an undesirable effect in the treatment of Class II malocclusion. 

Restriction of forward maxillary growth is one of the objectives of the headgear used to treat Class II malocclusions.^5^^,^ 6 ^,^ 16 In the present study, there was a reduction in the SNA angle between T_1_ and T_2_ only in Group 2 (CH + RME). However, comparison between groups did not show significant differences. Likewise, Üçem and Yüksel did not report effects over the SNA angle when combined headgear was used alone.^22^One can consider that the greater restriction in forward maxillary growth observed in subjects treated by rapid maxillary expansion is related to the distribution of forces over the maxilla provided by the connection of maxillary molars and premolars to the expander's acrylic plate and due to marked mobility caused by sutures separation.^10^

Clockwise rotation of the maxilla was observed between T_1_ and T_2_ in Group 1 (CH), but without significant difference from that found in Group 2 (CH+RME). The clockwise rotation of the maxilla is related to the direction of forces applied over maxillary molars. As molars are located in the posterior region of the arch, they can rotate the palatal plane and tilt the occlusal plane.^14^ This effect is undesirable, especially in patients with excessive exposure of gingival tissues and deep bite.^14^ According to O'Reilly et al,^17^ clockwise rotation of the maxilla also happens in Class II patients treated with cervical headgear; and according to Üçem and Yüksel,^22^ clockwise rotation may also be observed in patients treated with combined headgear. Therefore, it seems that only high-pull headgears would prevent or at least reduce maxillary clockwise rotation, based on a system of forces in which the resultant force passes through or above the center of resistance of the maxilla.^14^

Treatment effect can be considered equivalent to changes in the treated group minus changes in the control group. Comparison of mean differences (T_2_ - T_1_) between groups, as disclosed in Table 4, depict the main results of our study.

There was distal movement of maxillary molars in both treated groups when compared to the control group. On the other hand, distal tipping of molars was found only in Group 1 (CH). This finding is in agreement with those reported by Üçem and Yüksel.^22 ^Clockwise rotation of the maxilla was also considered a treatment effect of combined headgear used alone, based on the significant difference with the control group. This undesirable behavior could be expected, since it was previously found by O'Reilly et al^17^ and by Gautam et al.^14^

Based on these results, we consider that combined headgear, used alone or in association with rapid maxillary expansion, is an effective strategy as the first step of Class II malocclusion treatment. Additionally, rapid maxillary expansion seems to reduce initial treatment time, probably due to anterior accommodation of the mandible and favorable environment to anteriorposterior mandibular growth after expansion.^8^^,^ 9 ^,^ 11 Furthermore, deciduous molars and premolars are distally tipped together by their connection with the Haas expander, which reduces time and prevents a second phase of treatment. The clinical findings provided by this study allow the authors to recommend maxillary expansion before headgear appliance used to treat Class II associated with transverse maxillary deficiency. Further investigation, including final records (T_3_), should be carried out to provide better information about this treatment strategy.

## CONCLUSION

Combination headgear used as the first step of Class II malocclusion treatment results in the following:

» Distal movement of maxillary molars whether the headgear is used alone or in association with RME.

» Distal tipping of maxillary molars when the headgear is used alone: Group 1 (CH).

» Clockwise rotation of the maxilla when used alone: Group 1 (CH).
